# P-785. Construction and Validation of a Clinical Diagnostic Model for Active Tuberculosis in Patients with Systemic Lupus Erythematosus

**DOI:** 10.1093/ofid/ofae631.979

**Published:** 2025-01-29

**Authors:** Lifan Zhang, Lidan Zhao, Yuchen Liu, Xiaochun Shi, Xiaoqing Liu

**Affiliations:** Peking Union Medical College Hospital, Beijing, Beijing, China; Peking Union Medical College Hospital (CAMS), Beijing, Beijing, China; Peking Union Medical College Hospital (CAMS), Beijing, Beijing, China; Peking Union Medical College Hospital, Chinese Academy of Medical Sciences and Peking Union Medical College, Beijing, Beijing, China; Peking Union Medical College Hospital, Chinese Academy of Medical Sciences and Peking Union Medical College, Beijing, Beijing, China

## Abstract

**Background:**

Systemic Lupus Erythematosus (SLE), an autoimmune disease, involves multiple immune dysfunctions. The progression of M.tb infection is closely related to the host's immune status. Abnormal immune functions increase the risk of active tuberculosis (ATB), particularly in patients with autoimmune diseases. Meanwhile, both SLE and ATB’s clinical symptoms includes several overlaps, resulting in a diagnosis challenge when without etiological evidence.

Forest plot showing the results of univariate logistic regression in the discovery cohort

**Figure:**
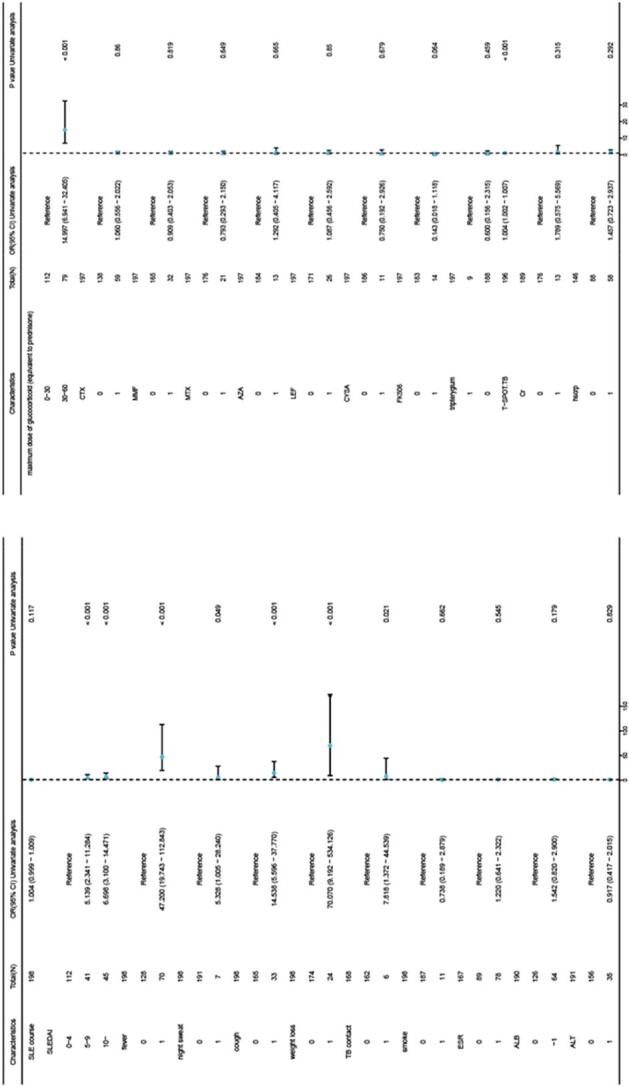

**Methods:**

We included 66 SLE patients with ATB hospitalized at Peking Union Medical College Hospital from 1975 to 2017, and 132 age matched SLE patients without ATB as the discovery cohort. An external validation cohort was drawn from the ETHERTB cohort, which covers rheumatic and immune disease patients from 13 tertiary hospitals in China. Univariate Logistic regression analysis was used in the discovery cohort to identify risk factors for ATB among SLE patients. A neural network algorithm was used to construct the clinical diagnostic model. The external validation cohort was used to validate the diagnostic potential of our model.

models developed

**Figure:**
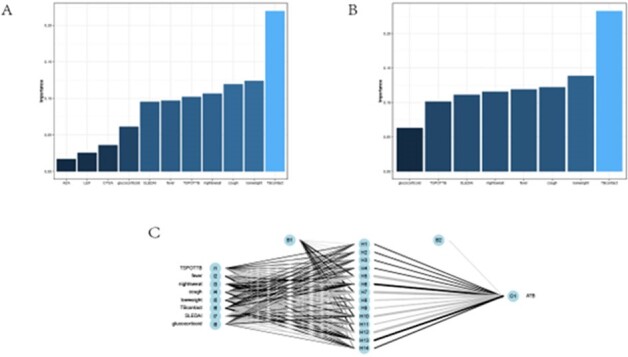

Importance of all risk factors (A) and variables included in our model (B). Neural network-based model architecture (C).

**Results:**

In the discovery cohort, fever (OR=47.2), night sweats (OR=5.3), cough (OR=14.5), weight loss (OR=70.1), moderate to severe SLE disease activity (SLEDAI-2000 5-9, OR=5.1; SLEDAI-2000≥10, OR=6.7), close contact with TB (OR=7.8), T-SPOT.TB (OR=1.004), and the use of specific immunosuppressants such as azathioprine (AZA) (OR=3.4), leflunomide (LEF) (OR=2.7), cyclosporine A (CYSA) (OR=3.8), and a maximum daily dose of 30 mg of corticosteroids (OR=14.9) were identified as risk factors for ATB in SLE patients. Variables including fever, night sweats, cough, weight loss, TB contact, T-SPOT.TB, SLEDAI-2000, and maximum daily corticosteroids dose were included in the diagnostic model based on an importance threshold of 0.5. The model's AUC was 0.96(95%Cl: 0.927-0.996), with a sensitivity of 85.3%, specificity of 96.0%, and an accuracy of 93.7%. In the external validation cohort, the AUC was 0.88(95Cl: 0.800-0.963), sensitivity 84.4%, specificity 88.1%, and accuracy 88.0%.

Evaluation of diagnosis model

**Figure:**
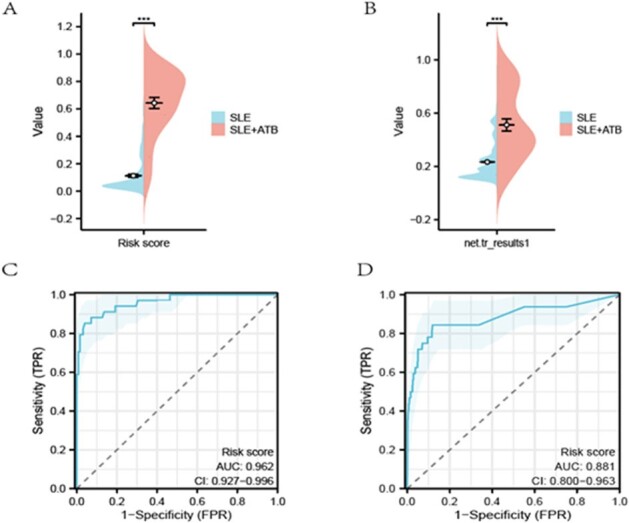

Distribution of risk scores in the discovery cohort (A) and validation cohort (B), and ROC curves in the discovery (C) and validation cohorts (D).

**Conclusion:**

Our study constructed and validated a diagnostic model for ATB in SLE patients. The model demonstrated good diagnostic potential, which could assist in diagnosis in the absence of etiological evidence.

**Disclosures:**

**All Authors**: No reported disclosures

